# Orbicularis oris muscle reconstruction and cheiloplasty with Z-plasty in a patient with a transverse facial cleft

**DOI:** 10.1186/s40902-019-0240-2

**Published:** 2019-12-02

**Authors:** Sung-Hyuk Koh, Yeon-Woo Jeong, Jeong Joon Han, Seunggon Jung, Min-Suk Kook, Hee-Kyun Oh, Hong-Ju Park

**Affiliations:** Department of Oral and Maxillofacial Surgery, School of Dentistry, Dental Science Research Institute, Chonnam National University, 42, Jebong-ro, Dong-gu, Gwangju, 61469 South Korea

**Keywords:** Transverse facial cleft, Hemifacial microstomia, Orbicularis oris muscle, Cheiloplasty

## Abstract

**Background:**

Transverse facial clefts are Tessier’s number 7 facial cleft among numbers 1–15 in Tessier’s classification of craniofacial malformations, which varies from a simple widening oral commissure to a complete fissure extending towards the external ear.

**Case presentation:**

In a patient with a transverse facial cleft, to functionally arrange the orbicularis oris muscle and form the oral commissure naturally, we performed a surgical procedure including orbicularis oris muscle reconstruction and cheiloplasty with Z-plasty.

**Conclusion:**

We achieved good results functionally and esthetically by orbicularis oris muscle reconstruction and cheiloplasty with Z-plasty. The surgical modality of our anatomical repair and 3 months follow-up results are presented.

## Background

Transverse facial cleft is a rare congenital anomaly. It may occur in combination with other systemic diseases or as an isolated condition, resulting from the lack of ectomesenchyme formation or penetration of the maxillary and mandibular processes during the fourth and fifth weeks of development. The defective area encompasses the commissure from the angle of the mouth to the cleft of the intraoral mucosa and buccal skin. The deep muscles appear to be split, with the buccinator and masseter muscles diverging unilaterally or bilaterally. In some severe cases, the cleft continues up to the zygomaticus major and minor muscles, rupturing the upper buccal region. As for the lower lip region, the cleft may involve the risorius muscle. It is also referred to as macrosomia, because of the appearance of a relatively big mouth, extending towards the ear.

According to Tessier’s classification of orbital/facial clefts, Tessier’s number 7 indicates temporo-zygomatic clefts found in the Treacher Collins syndrome or hemifacial microsomia. Transverse facial clefts are associated with anomalies of the external auditory meatus, middle ear, temporalis muscle, and seventh cranial nerve, and skeletal malformations, including lateral facial clefts.

Transverse facial clefts are more common in men than in women. The prevalence varies based on the statistics, but the estimated incidence is 1 in 80,000 live births [1]. Transverse facial clefts can occur unilaterally or bilaterally. It can be accompanied with other disorders, such as hemifacial microsomia, dwarfism, necrotic facial dysplasia, otomandibular dysostosis, unilateral facial agenesis, branchiogenic deformity, and first and second branchial arch syndromes [2].

Transverse facial clefts are considered to have multifactorial inheritance, including a combination of hereditary and non-hereditary causative factors. Prenatal ultrasound examination is used for early diagnosis of transverse facial clefts. However, it is difficult to obtain an early diagnosis in cases of bilateral microform clefts [3].

The repair of a transverse facial cleft has the following objectives: to achieve symmetrical oral opening, to make the commissure appear natural, to maintain function of the orbicularis oris muscle, to minimize external scarring, and to avoid lateral commissural migration [4]. For reconstruction of the natural appearance of the commissure, we utilized the Z-plasty technique, in which the outline of the incision is located in the skin, and a mucocutaneous flap is raised inferiorly. Here, we discuss the importance of this operative technique for management of transverse facial cleft.

## Case presentation

**Fig. 1:**
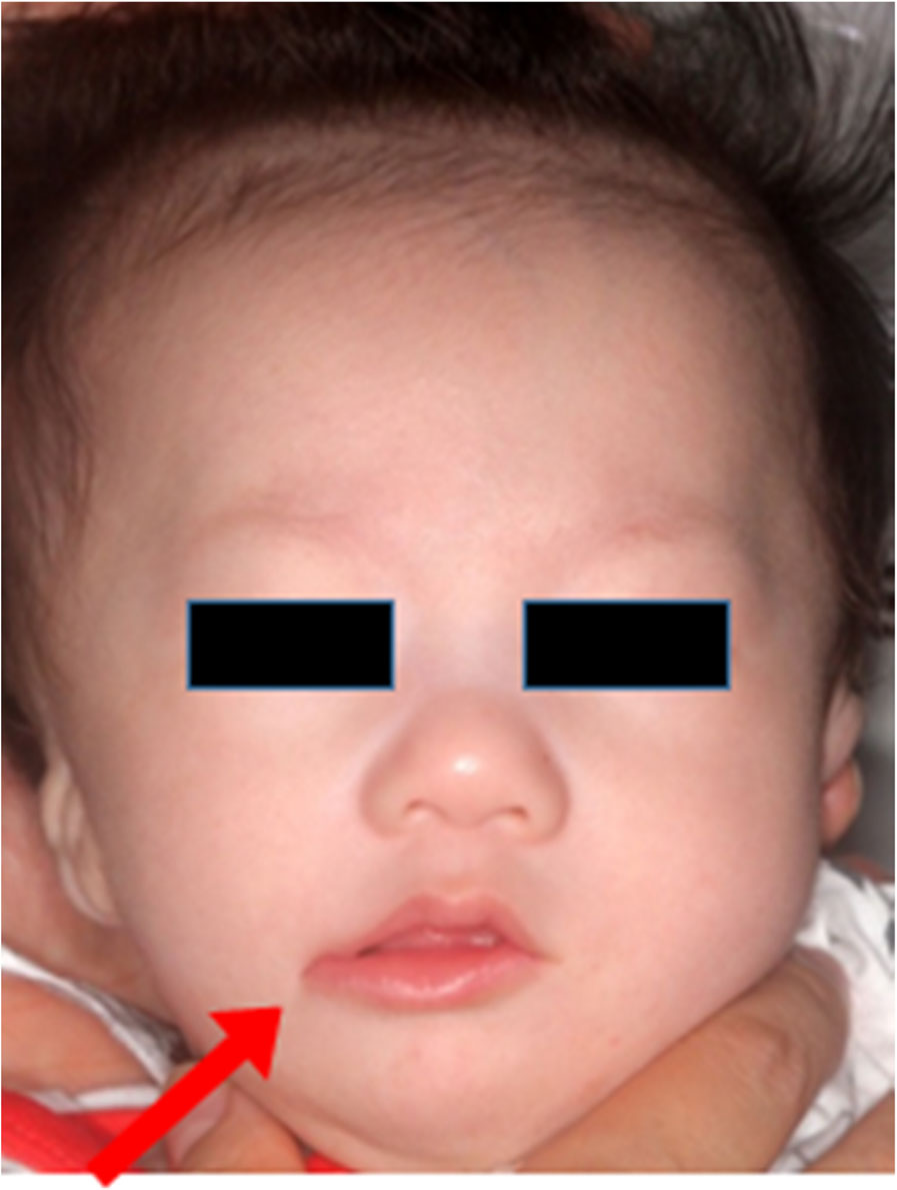
Preoperative photograph of the patient with a right transverse facial cleft

The patient was a 5-month-old boy, delivered through cesarean section on March 7, 2017. He was brought to our department on April 24, 2017, and was diagnosed as having a right transverse facial cleft with an incomplete cleft palate. Further, we delivered the Hotz appliance. Figure 1 shows the right transverse facial cleft. The patient had Goldenhar syndrome as a systemic disease. And on our clinical examination, the patient had a cleft in the right corner of the mouth, macrostomia, malposition of the orbicularis oris muscle, and right oral commissure, which was pulled laterally and downwards.

**Fig. 2:**
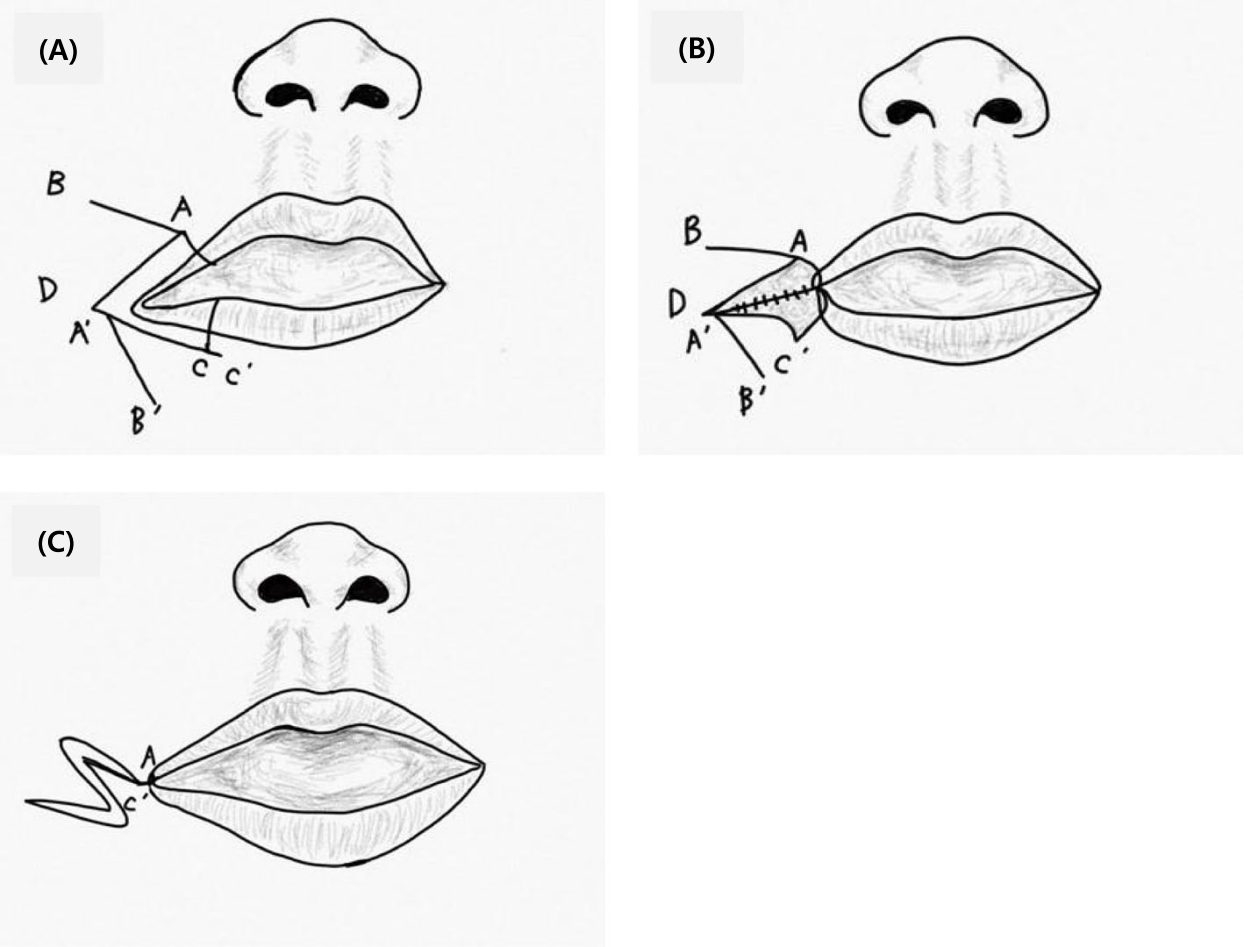
Markings for repair of the transverse facial cleft. **a** Point A is located on the healthy side. Point C is located on the commissure side to raise mucocutaneous flap. Incision is made at point C′. **b** Perpendicular incisions are made through the vermilion border of the lip. Incisions A–D and C′–D are made along the lip, beginning medially and continuing up to point D. **c** A mucocutaneous flap is elevated from the lower lip and sutured to the upper lip to create a new oral orifice. Points A and C′ are joined to create the white lip in the region of the commissure. Z-plasty flaps are sutured completely

**Fig. 3:**
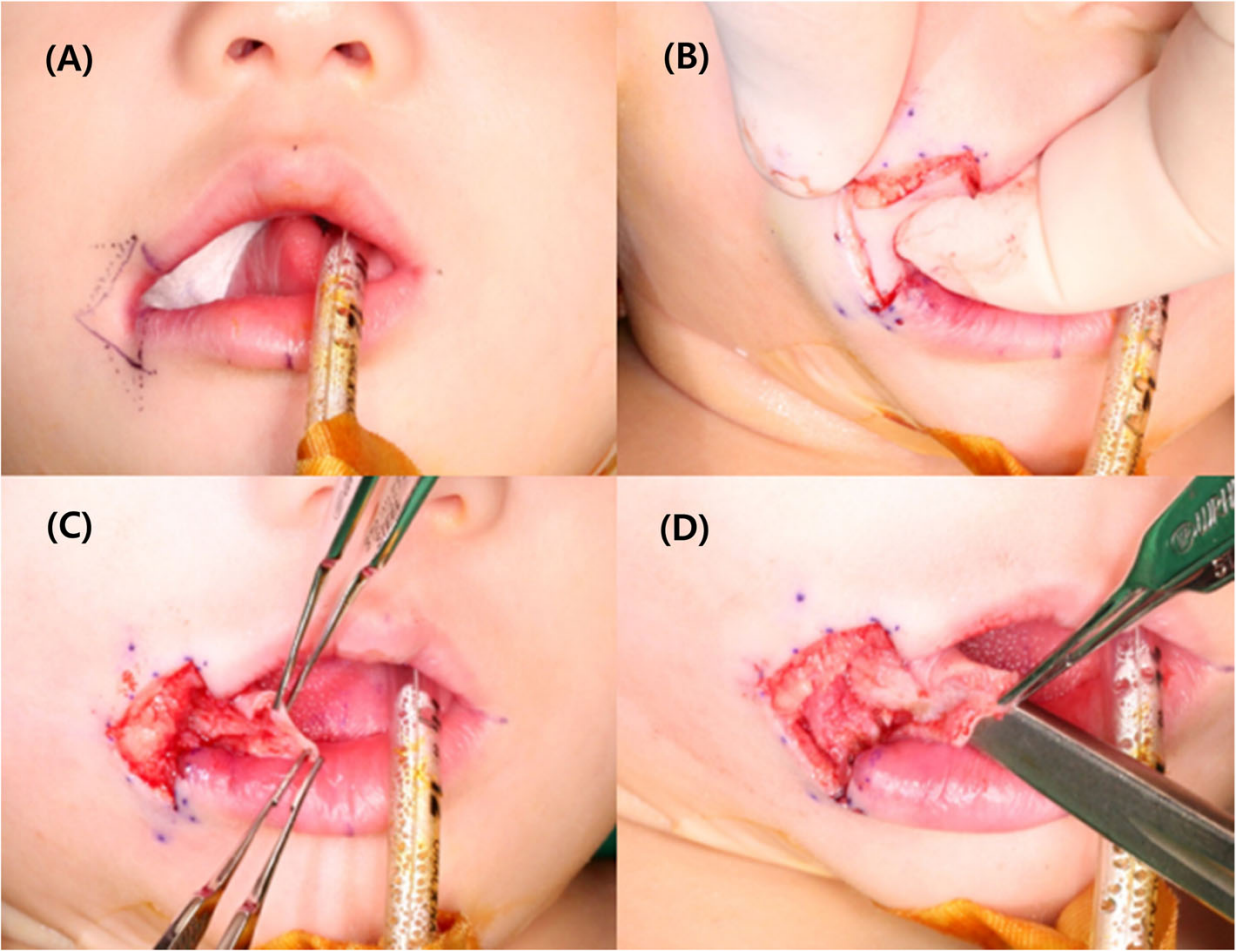
Intraoperative photographs. **a** The incision is designed. **b**, **c** Incisions are made in both extraoral and intraoral cleft regions. **d** The mucocutaneous flap is raised

**Fig. 4:**
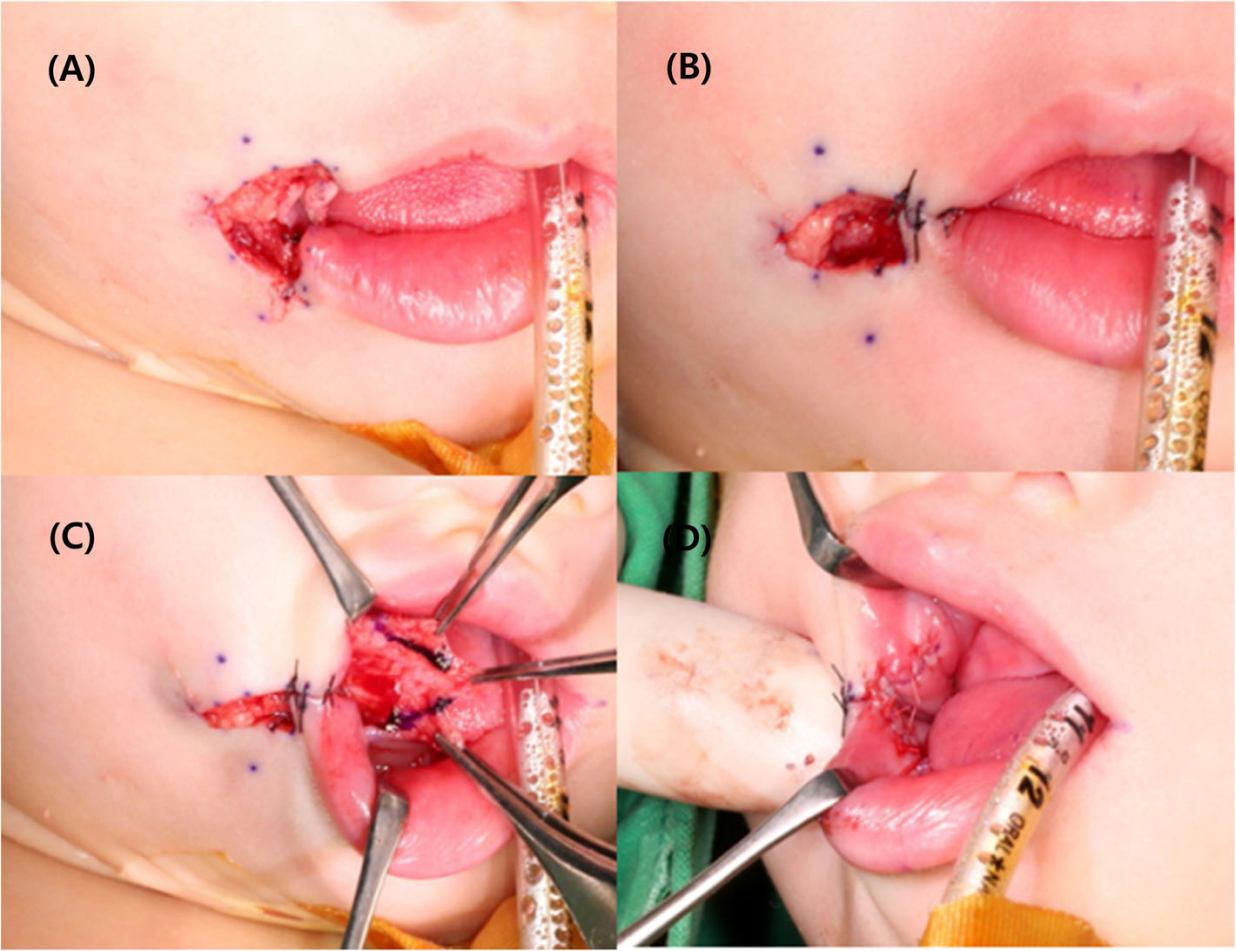
Intraoperative photographs. **a**, **b** Extraoral primary closure is performed. **c** An additional incision is made on the intraoral mucosal flap. **d** The bucco-mucosal cleft is closed

**Fig. 5:**
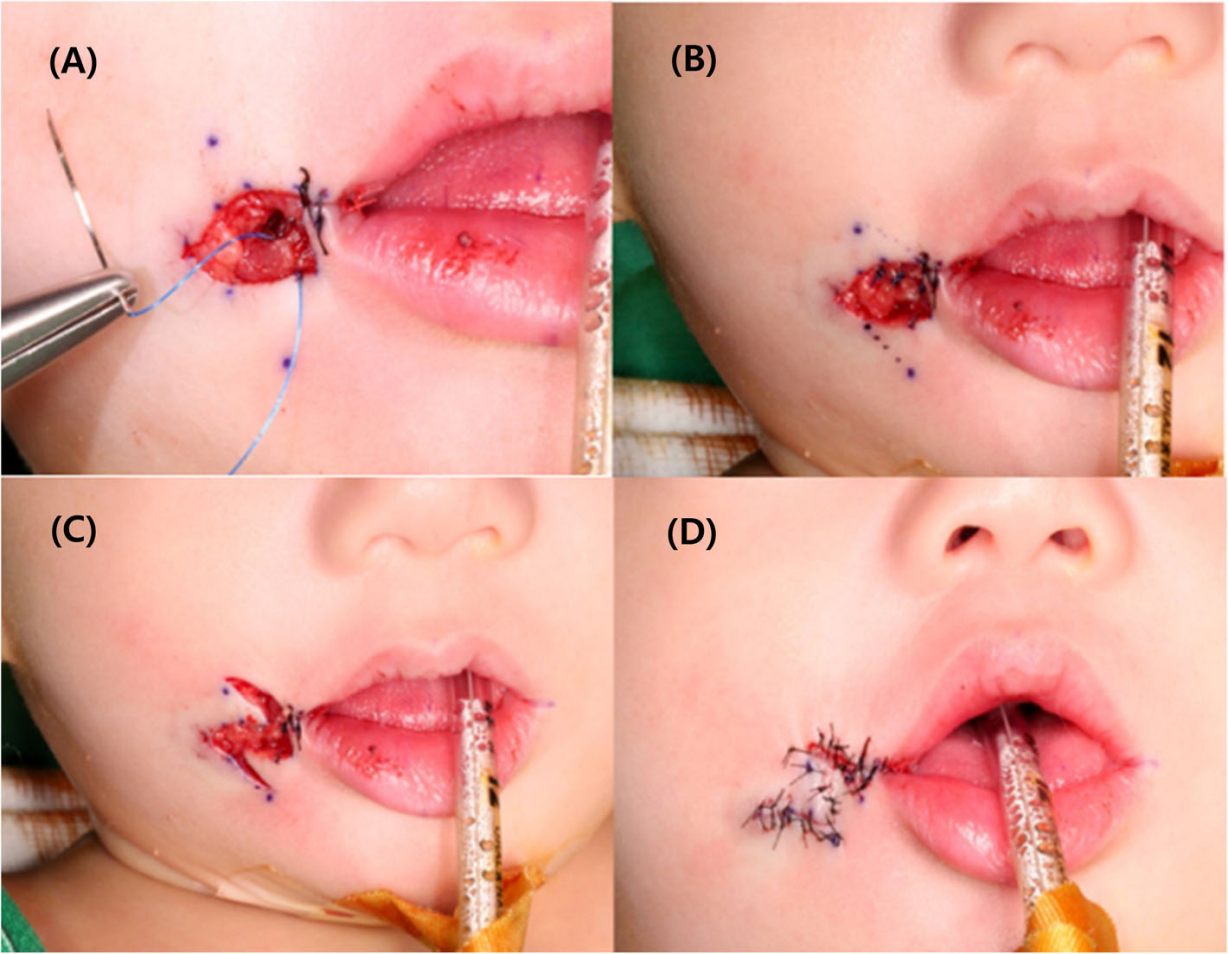
Intraoperative photographs. **a** Dissection of the muscle layer and exposure of the orbicularis oris muscle are performed. **b** The final muscle closure is performed. **c**, **d** The final Z-plasty surgical technique is used

Orbicularis oris muscle reconstruction and cheiloplasty using a mucocutaneous flap and Z-plasty were performed. Figure 2 shows the operative technique for reconstruction of the transverse facial cleft. The general operation technique was conducted following the method performed by Dr. Akita for reconstruction of a transverse facial cleft [5]. First, an incision was made using the healthy side as reference. To raise a mucocutaneous flap, incisions were made both extraorally and intraorally (Fig. 3). Extraoral primary closure was performed for the newly formed oral orifice. To avoid dysfunctions, such as those of mouth opening, pronunciation, and mastication, an additional incision was made on the intraoral mucosal flap, and the bucco-mucosal cleft was closed (Fig. 4). The muscle layer and exposed orbicularis oris muscle were dissected. To reconstruct the modiolus region, the inferior part of the orbicularis oris muscle was overlapped with its superior part, and muscle closure was performed. Subsequently, the mucosa was closed with the Z-plasty technique to prevent wound contraction and obtain a good facial profile in the patient (Fig. 5).

**Fig. 6:**
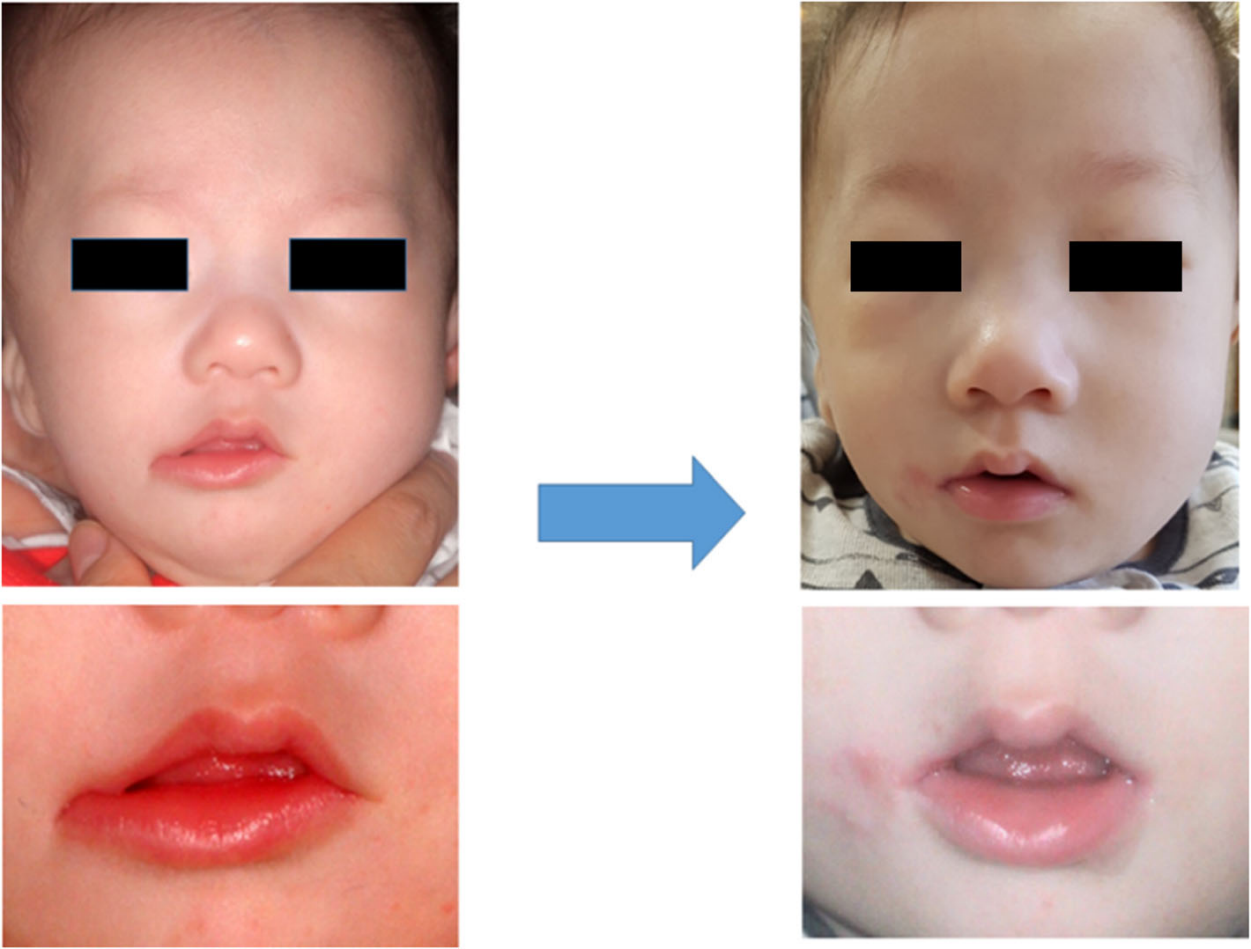
Preoperative photographs (left) and photographs after 3 postoperative months (right)

Figure 6 shows the photographs acquired before and 3 months after the surgery. At 3 postoperative months, symmetry was observed between both the oral commissures with satisfactory esthetic reconstruction, and there were no functional postoperative complications.

## Discussion

The transverse facial cleft has been called the lateral facial cleft, lateral facial commissure, and macrostomia because of the widening of the mouth with a cleft extending up to the ear. In severe cases, the buccal cleft reaches up to the anterior region of the ear, and the mouth appears to be wide antero-posteriorly, with clinical findings, such as preauricular tags [3]. Transverse facial clefts are more common in men than in women. The prevalence varies according to statistics, but the incidence is approximately 1 in 80,000 live births, and approximately 10–20% of the cases of transverse facial clefts are bilateral, which represents about 5.5% of the cases based on the Tessier classification [6–8]. Tessier’s number 7 clefts have been reported in only a few instances in the Korean literature, and the incidence of transverse or lateral facial cleft is 0.45% [9].

According to Tessier’s classification of orbital/facial clefts, Tessier’s number 7 indicates temporo-zygomatic clefts found in the Treacher Collins syndrome or hemifacial macrosomia [10]. Transverse clefts are associated with anomaly in the external auditory meatus, middle ear, temporalis, and seventh cranial nerve; hair anomalies in the anterior region of the ear; and skeletal malformations, including lateral facial clefts that cause mandibular posterior alveolar hypoplasia at the pterygomaxillary junction. In cases of the defect affecting only the soft tissue, the cleft starting from the corner of the mouth runs supero-laterally towards the upper buccal region, reaching the anterior region of the ear. In such cases, the lower eyelid and external auditory meatus are normal, and there are no scars in the region of the ear. Transverse facial cleft have hypoplastic coronoid process in the mandible, asymmetrical cranial base, and tilted/asymmetric temporomandibular joint. Thus, Tessier’s number 7 facial cleft is an orbital/facial classification including clefts involving the hard tissue and accompanied with Tessier’s number 6 and 8 facial clefts, Treacher Collins syndrome, and the Goldenhar syndrome [11–15].

Transverse facial clefts can only be treated surgically to obtain a normal appearance and to restore speech and masticatory functions in the patient. If the cleft is restricted to the mucosal soft tissues, it is possible to incise the border of the cleft region and, subsequently, perform suturing in layers. However, in the standard procedure, the unfused orbicularis oris muscles of the upper and lower lips are placed in proximity and sutured closed. There are several reports on the time of the surgery [6, 16–21]. It is advisable to perform cleft lip and palate surgery at the earliest after 3 months of age. Further, an anatomical or functional approach to rebuild the cleft region is important.

Numerous techniques have been proposed to construct the commissure in cases of a transverse facial cleft (Table 1). Early studies employed a straight-line closure of the vermilion border and intraoral mucosa [32]. However, placing the scar at the commissure often resulted in fissuring, contracture, and an unnatural appearance [22–24]. Based on the principles used in oral reconstruction of an electrical burn scar [36], other surgeons began to use a vermilion-mucosal flap to line the commissure. Both superiorly [29, 33] and inferiorly [1, 19, 23, 24, 28, 32, 34, 35] based vermilion-mucosal flaps have been described. However, as Eguchi et al. reported, the insertion scar on the lower lip, with raising a superiorly based flap, is more conspicuous with oral opening compared to raiding inferiorly based flap [24]. In this case, we prefer an inferiorly based rectangular vermilion-mucosal flap to form the commissure for the following reasons: (1) the insertion scar in the upper lip is inconspicuous, (2) the color and thickness of the vermilion-mucosal flap are normal, and (3) the commissure changes shape in a normal fashion during oral opening and repose [4].

**Table 1:** Techniques for repair of transverse facial cleft

Cutaneous closure	Commissural closure					
	Linear	Vermilion-mucosal flap/inferiorly based	Vermilion-mucosal flap/ superiorly based	Vermilion-mucosal-cutaneous flap	Commissure transposition	Cutaneous triangular flap
Linear	Blackfield and Wilde [22]	Powell and Jenkins [23] Eguchi et al. [24]		Nagai and Weinstein [25] Weinstein [26]		Kawai et al. [18]
Z-plasty	Boo-Chai [27]	Chen and Noordhoff [28]	Kaplan [29]	Aketa et al. [5]	Fukuda and Takeda [22]	Onizuka [30] Yoshimura et al. [16] Ono and Tateshita [31]
W-plasty	Habal and Scheuerle [32]	Habal and Scheuerle [32]	Itho et al. [33] Bauer et al. [34]	May [35]	Fukuda and Takeda [22]	

Suturing of the intraoral mucosa should be performed first. Subsequently, the upper and lower buccal skin are sutured. Although intraoral mucosal sutures are less noticeable in the external scar or function, and various suture methods may be considered, the authors recommend the continuous locking suture method. For children, a 4–0 or 5–0 absorbable suture is used, so that the knot in the suture is released into the mouth to prevent transparency and interference with the buccal muscles and skin. In addition, the knot should be placed in the oral cavity to be stitched out or self-absorbed, and in particular, the suture knot should never be exposed in the labial commissure region.

In the process of suturing the upper and lower orbicularis oris muscles, as the deep muscle layer runs horizontally in the mucosa of the oral cavity, the sutures starting from the mucosa should always be everted so that a round shape of the mouth is formed. As the superficial muscles are finely divided into the surrounding buccinator muscle in the skin, they should not be too thick when holding the muscle, and 5–0 or 6–0 sutures should be used, so that the fascia can meet. In particular, when suturing the lower orbicularis oris, the buccal pad under the fascia should delicately be undermined and exposed, so that the buccal skin at the longest distance from the labial commissure is not thickened without tension. Furthermore, the upper and lower fascia should meet, so that the buccal skin appears natural. When postoperative scarring occurs as the skin suture is proceeded, Z-plasty can be performed in the region where the upper and lower red vermilion borders meet. It can be accomplished whenever necessary to place the postoperative scar of the lip and lip commissure to the region of wrinkled skin area. This is more important in pediatric patients. As the patient grows, with reduction in tension between the upper and lower orbicularis oris muscles, we can achieve a natural looking modiolus through formation of the labial commissure ring.

It is important to set standards for the position of the labial commissure in the repair of transverse facial clefts. In unilateral cases, the normal side of the labial commissure becomes a good standard, but in bilateral cases, it is difficult to set standards. In such cases, by examining the transition of the vermilion border and the buccal mucosa and assessing the connected region, we can determine the position of the labial commissure. As described before, the muscular fibers of the deep and superficial orbicularis oris muscles should form a vertical relationship with each other at the labial commissure, and if not, it has been reported that a “goldfish mouth” like wrinkle can be formed because of the lack of muscles in the labial commissure [27].

## Conclusion

The transverse facial cleft is a type of congenital facial anomaly that occurs rarely in Korea as mentioned above. To achieve normal mastication, pronunciation, and swallowing, an appropriate surgical approach is essential, and the oral and maxillofacial surgeons should be familiar with the clinical technique. It is desirable to perform at the earliest, considering the physical status of the patient. As repositioning of the intraoral mucosa and of the orbicularis oris muscle is crucial, it is necessary to learn various approaches for skin incisions. In this transverse facial cleft case, we have performed a surgical procedure using advanced orbicularis oris muscle reconstruction and cheiloplasty with Z-plasty. As a result, we obtained symmetry of both oral commissure and esthetically good results.

## Data Availability

Data sharing not applicable to this article as no datasets were generated or analyzed during the current study.
